# The IRE1α Pathway Links Endoplasmic Reticulum Stress to Atherosclerosis‐Related Inflammation and Lipid Accumulation

**DOI:** 10.1155/mi/4439938

**Published:** 2026-04-08

**Authors:** Mariam Bagheri Ekta, Natalia Elizova, Stanislav Antonov, Alexander Orekhov, Vasily Sukhorukov

**Affiliations:** ^1^ Laboratory of Cellular and Molecular Pathology of the Cardiovascular System, Petrovsky National Research Center of Surgery (Formerly Russian Scientific Center of Surgery), Abrikosovsky Lane 2, Moscow, 119991, Russia; ^2^ Laboratory of Molecular Genetic Modeling of Inflammaging, Institute of General Pathology and Pathophysiology, Baltiyskaya Street 8, Moscow, 125315, Russia, niiopp.ru

**Keywords:** atherosclerosis, CRISPR/Cas9, inflammation, IRE1α, macrophages, unfolded protein response

## Abstract

Endoplasmic reticulum stress (ER stress) is closely related to the pathogenesis of atherosclerosis through various mechanisms, including inflammatory responses and foam cell formation. However, the mechanisms by which ER stress contributes to atherosclerosis require further elucidation. In this study, we investigate the impact of the inositol‐requiring enzyme 1 alpha (IRE1α) arm of the unfolded protein response (UPR) in the expression of inflammatory cytokines in monocytes and intracellular lipid accumulation in macrophages, which play a crucial role in the immune response associated with atherosclerosis. We created an IRE1α knockout (KO) THP‐1 monocytic cell line using the CRISPR/Cas9 gene‐editing technology and subsequently differentiated these cells into macrophages. We conducted a comparative analysis of IRE1α KO cells and control THP‐1 cells, focusing on several parameters: morphological features, lipopolysaccharide (LPS)‐induced proinflammatory cytokine responses, specifically interleukin‐1 beta (IL‐1β), interleukin‐6 (IL‐6), and tumor necrosis factor (TNF) α measured by quantitative real‐time PCR (qPCR) and enzyme‐linked immunosorbent assay (ELISA), as well as intracellular cholesterol accumulation and the expression levels of CD36 and ABCA1 genes following exposure to low‐density lipoproteins (LDLs) derived from patients with atherosclerosis. Our findings demonstrate that IRE1α KO resulted in significant reduction of TNF, IL‐1β, and IL‐6 expression following LPS stimulation (*p*  < 0.05). ELISA confirmed significantly reduced cytokine secretion in IRE1α KO monocytes compared to controls. Furthermore, IRE1α deficiency impaired the cellular response to atherogenic LDL, preventing lipid‐induced upregulation of scavenger receptor CD36 and cholesterol efflux transporter ABCA1. Thus, IRE1α serves as a critical regulator of both inflammatory cytokine expression and lipid metabolism in THP‐1 cells, highlighting its potential as a therapeutic target for inflammatory diseases and atherosclerosis. Targeting IRE1α could offer new strategies to address inflammation and lipid dysregulation in cardiovascular diseases.

## 1. Introduction

Atherosclerosis is a chronic inflammatory disease characterized by the accumulation of lipids, inflammatory cells, and fibrous elements within the arterial wall, leading to the formation of atherosclerotic plaques [1]. Macrophages play a pivotal role in the pathogenesis of atherosclerosis, where they contribute to both the inflammatory response and lipid metabolism [2]. The regulation of inflammatory cytokines is crucial in modulating macrophage activation and the progression of atherosclerosis [3]. Among the various signaling pathways involved in proinflammatory macrophage activation, the unfolded protein response (UPR) plays a significant role [4, 5].

The inositol‐requiring enzyme 1 alpha (IRE1α) has emerged as a significant player in the UPR and innate immune signaling. It is known as the most ancient branch of the UPR signaling pathway, being evolutionarily conserved from yeast to humans [6]. The IRE1 gene exists in two primary isoforms: IRE1α and IRE1β, which are encoded by the genes ERN1 and ERN2, respectively. IRE1α, the more widely studied isoform, is expressed in various tissues and plays a pivotal role in maintaining cellular homeostasis by regulating protein folding and secretion. In contrast, IRE1β is predominantly expressed in specialized epithelial cells, such as those in the airway and intestinal mucosa [7, 8].

IRE1α is a transmembrane protein that acts as a sensor of endoplasmic reticulum stress (ER stress), which can be triggered by various stimuli, including lipid accumulation and inflammatory signals [9, 10]. Upon activation, IRE1α undergoes dimerization and autophosphorylation, activating its RNase activity to splice X‐box binding protein 1 (XBP1) mRNA, generating the active transcription factor XBP1s [11]. Beyond its canonical UPR functions, IRE1α has emerged as a crucial regulator of inflammatory signaling through its interaction with tumor necrosis factor (TNF) receptor‐associated factor 2 (TRAF2), leading to activation of nuclear factor kappa B (NF‐κB) and c‐Jun N‐terminal kinase (JNK) pathways [12, 13]. Several studies have demonstrated that IRE1α activation can enhance proinflammatory cytokine production in various cell types [14, 15]. In macrophages, IRE1α has been implicated in Toll‐like receptor (TLR) signaling enhancement and inflammasome activation [16, 17]. Furthermore, metabolic stress conditions, including lipid overload commonly observed in atherosclerosis, can trigger IRE1α activation and subsequent inflammatory responses [18, 19]. Despite the established role of IRE1α in regulating ER stress and inflammation, its specific impact on cytokine expression and lipid accumulation in macrophages within the context of atherosclerosis remains incompletely understood.

In our previous study, we found that the knockdown of ER stress gene PERK significantly reduced cholesterol accumulation in monocytes isolated from the peripheral blood of apparently healthy individuals [20]. Considering that UPR is mediated through three branches—PERK, IRE1α, and ATF6—that often exhibit compensatory and synergistic effects, these results raise an important question about the contribution of the IRE1α pathway to lipid dysregulation. Furthermore, since ER stress is linked to the development of both metabolic dysfunction and inflammation, we hypothesized that IRE1α functions as an important mediator linking cholesterol accumulation to proinflammatory activation in monocytes/macrophages. To systematically evaluate this hypothesis under controlled conditions, we utilized THP‐1 cells, a widely used and well‐characterized human monocytic cell line [21, 22]. This model offers several advantages over primary cells, including genetic homogeneity, unlimited availability, and reduced donor‐to‐donor variability [23, 24], while preserving key features of primary monocytes, such as functional stress responses [25, 26] and intact cholesterol metabolic pathways.

To address the knowledge gap regarding IRE1α’s role in inflammatory signaling in THP‐1 monocytes and lipid accumulation in THP‐1‐derived macrophages, we generated IRE1α knockout (KO) THP‐1 cells using CRISPR/Cas9 and assessed cytokine production in monocytes and lipid accumulation following macrophage differentiation. We hypothesized that IRE1α deficiency would significantly impair proinflammatory cytokine responses to lipopolysaccharide (LPS) stimulation, thereby providing insights into the molecular mechanisms underlying macrophage activation and identifying potential therapeutic targets for inflammatory diseases.

## 2. Materials and Methods

### 2.1. Cell Lines and Culture Conditions

Human monocytic leukemia cell line THP‐1 (human, peripheral blood, acute monocytic leukemia) was obtained from the Collection of Vertebrate Cell Cultures, Institute of Cytology, Russian Academy of Sciences, St. Petersburg in 2022. THP‐1, as well as the THP‐1 with IRE1α KO (THP‐1 IRE1α KO) cells, were cultured in a complete culture medium of RPMI‐1640 supplemented with L‐glutamine (Biowest), 10% (v/v) fetal bovine serum (FBS) (Gibco, Thermo Fisher Scientific, Inc.), 100 U/mL of penicillin−streptomycin (Gibco), and 50 μM β‐mercaptoethanol (Sigma–Aldrich) at 37°C under a humidified atmosphere with 5% CO_2_. The cells were maintained at the density of 10^6^ cells/mL with medium exchange every 2 days. THP‐1 was used as a control (reference) cell line. Cell lines were routinely tested for mycoplasma contamination using a PCR‐based assay (MycoReport, Evrogen) at regular intervals (every 4–6 weeks), and all tests performed during the period of the described experiments were negative. THP‐1 cells were authenticated for the described experiments by STR profiling (Institute of Cytology, Russian Academy of Sciences, St. Petersburg, 2022), showing *a* ≥ 98% match to the reference THP‐1 STR profile.

### 2.2. CRISPR/Cas9 KO Plasmid Transfection

The THP‐1 IRE1α KO cell line was generated using the IRE1α CRISPR/Cas9 KO plasmid vector (catalog # sc‐429758, Santa Cruz Biotechnology) via transfection, according to the manufacturer’s protocol CRISPR/Cas9 KO Plasmid Transfection (Santa Cruz Biotechnology). Single‐cell clones were selected and screened for IRE1α KO efficiency by quantitative real‐time PCR (qPCR).

### 2.3. Macrophage Differentiation

THP‐1 and THP‐1 IRE1α KO cells were differentiated into macrophage‐like cells by treatment with 100 ng/mL phorbol 12‐myristate‐13‐acetate (PMA, Sigma–Aldrich) for 48 h at 37°C.

### 2.4. CD68 Immunohistochemistry

THP‐1 and THP‐1 IRE1α KO cells were seeded on round coverslips and differentiated by the protocol described above. Cells were fixed with 4% buffered formaldehyde and stained with CD68 antibody (Abcam) following standard protocol.

### 2.5. Low‐Density Lipoprotein (LDL) Isolation and Treatment

LDL was isolated from plasma of patients with diagnosed atherosclerosis, including patients with myocardial ischemia of atherosclerotic origin, using standard ultracentrifugation protocols. A single pool of LDL was used for all experiments. THP‐1 and THP‐1 IRE1α KO cells, differentiated into macrophage‐like cells, were incubated with patient‐derived atherogenic LDL at a concentration of 100 μg/mL for 24 h to assess lipid‐induced responses and foam cell formation. Cell viability under these conditions was evaluated using the MTT assay and was not significantly affected (Figure S1).

### 2.6. Cell Viability Assessment

Cell viability was assessed using the MTT assay (Sigma–Aldrich). Briefly, cells were seeded at a density of 4 × 10^4^ cells per well in 96‐well plates and treated for 24 h with LPS (500, 1000, 1500, and 2000 ng/mL) and atherogenic LDL (100 μg/mL). In selected experiments, higher concentrations of LPS were used to evaluate dose‐dependent cytotoxicity. Following treatment, 20 μL of MTT solution (5 mg/mL in PBS) was added to each well, and cells were incubated for 2 h at 37°C. Formazan crystals were subsequently dissolved in DMSO, and absorbance was measured at 570 nm using a microplate reader. Cell viability was calculated as a percentage relative to untreated control cells.

### 2.7. RNA Extraction, cDNA Synthesis, and qPCR Analysis

Total cellular RNA of THP‐1 and THP‐1 IRE1α KO cells was extracted by magnetic bead‐based (Biolabmix) according to the manufacturer’s protocol. The RNA from each sample was then converted to cDNA using the M‐MuLV–RH kit (Biolabmix) according to the manufacturer’s protocol. qPCR was performed in triplicate using CFX96 Touch (BioRad) with 2x Hi‐ROX SYBR Green Master Mix (Biolabmix) and specific primers. mRNA levels were normalized to the GAPDH housekeeping gene. Relative expressions were calculated with the Pfaffl method. The primer sequences used were as follows: interleukin‐1 beta (IL‐1β) forward, 5^′^‐GCTCGCCAGTGAAATGATGG and reverse 5^′^‐GGTGGTCGGAGATTCGTAGC; interleukin‐6 (IL‐6) forward 5^′^‐CATCCCATAGCCCAGAGCAT and reverse 5^′^‐TGGGTCAGGGGTGGTTATTG; TNF forward, 5^′^‐TCCCCAGGGACCTCTCTCTA and reverse 5^′^‐CTTGTCACTCGGGGTTCGAG; IRE1α, forward 5^′^‐CGGCCTCGGGATTTTTGGAA and reverse 5^′^‐TTCCATCCAGCGTTGACACA; ABCA1, forward 5^′^‐GGGTCTGTCCCCAGCATAAC and reverse 5^′^‐GCTTGCTTGATGGCAAACCA; CD36 forward, 5^′^‐AACCACACACTGGGATCTGAC and reverse 5^′^‐CTGCAGGAAAGTCCTACACTG; GAPDH forward, 5^′^‐GTCAACGGATTTGGTCGTATTG and reverse 5^′^‐TGTAGTTGAGGTCAATGAAGGG.

### 2.8. LPS Stimulation and Enzyme‐Linked Immunosorbent Assay (ELISA) for IL‐1β, TNF, and IL‐6

For cytokine induction studies, THP‐1 and THP‐1 IRE1α KO monocytes were stimulated with 1000 ng/mL LPS from *Escherichia coli* O111:B4 (Sigma–Aldrich) for 24 h to induce proinflammatory responses. Culture supernatants from LPS‐stimulated THP‐1 and THP‐1 IRE1α KO monocytes were collected after 24 h of treatment. Concentrations of IL‐1β, TNF, and IL‐6 were measured using commercial ELISA kits (R&D Systems, Inc.) according to the manufacturer’s instructions.

### 2.9. Cellular Cholesterol Content Measurement

Cells were lysed in a 0.2 N sodium hydroxide solution. Protein levels were measured according to the Lowry method using 1 mg/mL bovine serum albumin as a standard. Cellular lipids were extracted from cell samples using hexane–isopropanol (3:2; v/v). Cholesterol levels were measured using Fluitest CHOL (Analyticon) with 1 mg/mL cholesterol solution as a standard. Total cholesterol content was calculated as the ratio of cholesterol to cell protein.

### 2.10. Morphological Analysis

THP‐1 and THP‐1 IRE1α KO cells were fixed with 4% paraformaldehyde for 20 min, washed twice with PBS, and stained with Giemsa solution (Sigma–Aldrich) for 10–15 min at room temperature. Morphological changes were observed using an inverted microscope. Images were analyzed using FIJI 1.52n software. Cell area was measured from manually selected macrophage‐like cells and is expressed in μm^2^. Giemsa‐stained coverslips were imaged with a Leica DM 2500 microscope using a 40x objective, and counts were done for at least three random fields. All brightfield images were taken using a Leica DM 2500 microscope.

### 2.11. Statistical Analyze

All experiments were performed in triplicate to ensure reproducibility. Data normality was assessed using Shapiro–Wilk or Kolmogorov–Smirnov tests. Differences between the experimental groups were analyzed using the Student’s *t*‐test or one‐way analysis of variance (ANOVA). Data are presented as the mean ± standard deviation (SD). Microsoft Office Excel 2020 (Microsoft, Inc.) and GraphPad Prism 8.0 software (GraphPad Software, Inc.) were used for statistical analysis. A *p*‐value < 0.05 was considered statistically significant.

### 2.12. Manuscript Clarification

For clarity, LPS‐induced cytokine mRNA/protein measurements were performed in undifferentiated THP‑1 monocytes, whereas lipid‐loading/foam‐cell assays were performed in PMA‐differentiated THP‑1 macrophage‐like cells (as specified for each experiment).

## 3. Results

### 3.1. IRE1α Gene KO Led to Morphological Changes of Monocyte and Macrophage‐Like THP‐1 Cells

**Figure 1:**
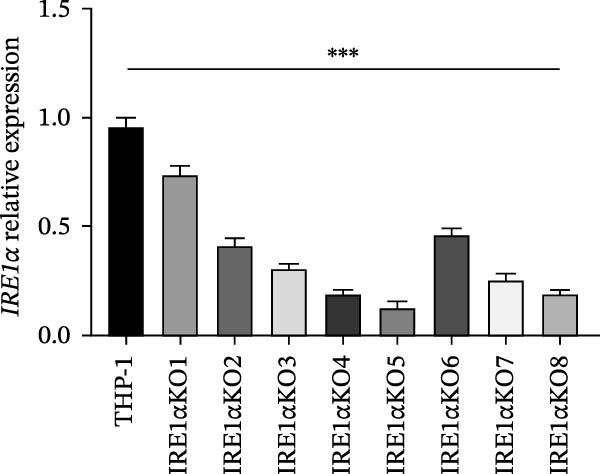
IRE1α gene expression in CRISPR/Cas9‐generated knockout clones. Data are presented as mean ± SD. Statistical significance: ns, not significant;  ^∗^ *p*  < 0.05;  ^∗∗^ *p*  < 0.001;  ^∗∗∗^ *p*  < 0.0001. One‐way ANOVA with Bonferroni correction. THP‐1, parental control cells; IRE1α KO1‐KO8, THP‐1 cell clones with IRE1α knockout.

As a result of transfection of the THP‐1 monocytic cell line with an IRE1α CRISPR/Cas9 plasmid, we obtained clones. These clones were subsequently evaluated for IRE1α gene expression using qPCR (Figure 1). Clone IRE1α KO5, which demonstrated >95% reduction in IRE1α mRNA expression compared to parental THP‐1 cells (Figure 1), was selected for subsequent experiments.

We found that IRE1α KO did not prevent the maturation process of THP‐1 macrophage‐like cells. Cells differentiated into macrophages expressed the macrophage‐associated marker CD68 at levels comparable to control cells (Figure 2A), indicating that macrophage differentiation, as assessed by CD68 expression, can occur in the absence of IRE1α. But despite this, IRE1α KO cells exhibited distinct morphological differences compared to control THP‐1 cells (Figure 2B). To determine whether these changes reflect incomplete differentiation or a distinct cellular phenotype, morphological parameters were quantified independently of differentiation status. IRE1α KO monocytic cells demonstrated significantly reduced cell area (median 36.9 μm^2^) compared to control THP‐1 cells (median 63.4 μm^2^; *p*  < 0.0001, Mann–Whitney *U* test) (Figure 2C). Following PMA‐induced differentiation, IRE1α KO macrophage‐like cells also exhibited a reduced cell area compared to controls (median 146.2 μm^2^ vs. 224.8 μm^2^, respectively; *p*  < 0.001) (Figure 2D). Notably, these size differences were observed both before and after differentiation, suggesting a stable alteration in cellular morphology rather than a transient delay in maturation. Macrophage differentiation efficiency, defined as the proportion of cells exhibiting a flattened morphology and vesicular cytoplasm, was reduced in IRE1α KO cells (28%) compared to control cells (60%) (Figure 2E; Figure S2). Importantly, cells in the IRE1α KO group that fulfilled morphological criteria for macrophage differentiation exhibited defining morphological features comparable to those of control macrophages.

Figure 2Morphological changes in THP‐1 and THP‐1 IRE1α KO cells during monocyte‐to‐macrophage differentiation. (A) CD68 immunohistochemistry of THP‐1 and THP‐1 IRE1α KO macrophage‐like cells; (B) Romanowsky–Giemsa staining of THP‐1 and THP‐1 IRE1α KO cells; (C) monocyte cell area measurements for THP‐1 and THP‐1 IRE1α KO cells; (D) macrophage cell area measurements for THP‐1 and THP‐1 IRE1α KO cells; (E) quantification of differentiated macrophages from THP‐1 and THP‐1 IRE1α KO cells, THP‐1 inter and IRE1α KO inter–intermediate forms between monocytes and macrophages. Data are presented as mean ± SD. Statistical significance:  ^∗^ *p*  < 0.05;  ^∗∗^ *p*  < 0.01;  ^∗∗∗^ *p*  < 0.001. Statistical analysis performed using Mann–Whitney *U* test.

**Figure:**
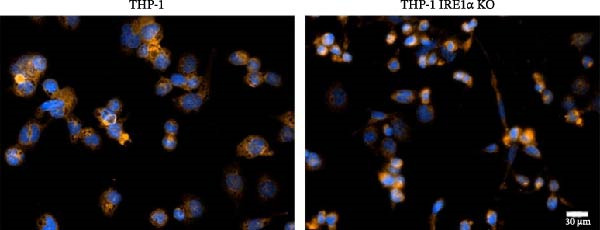
(A)

**Figure:**
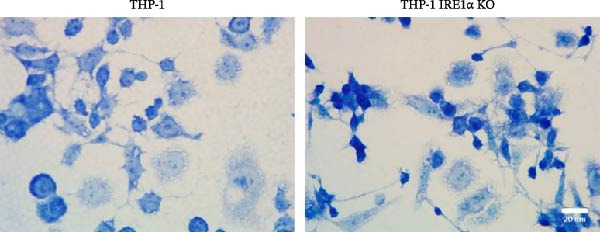
(B)

**Figure:**
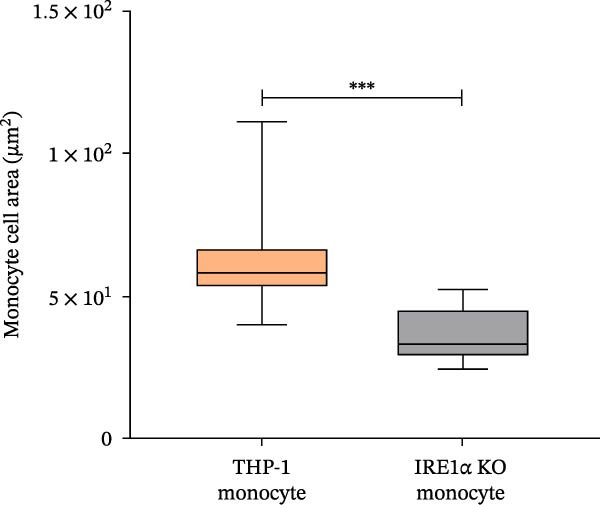
(C)

**Figure:**
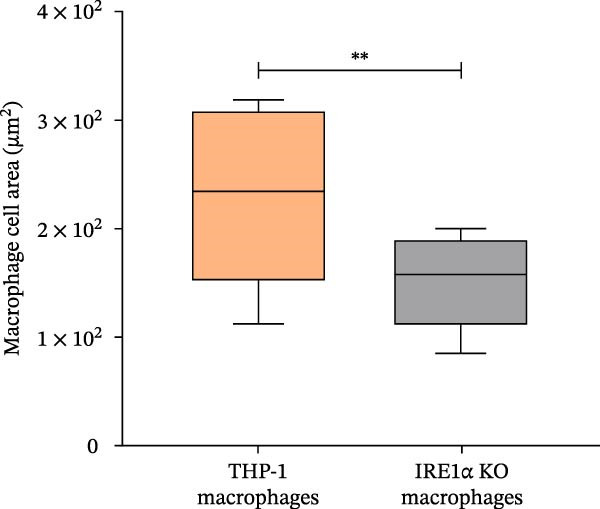
(D)

**Figure:**
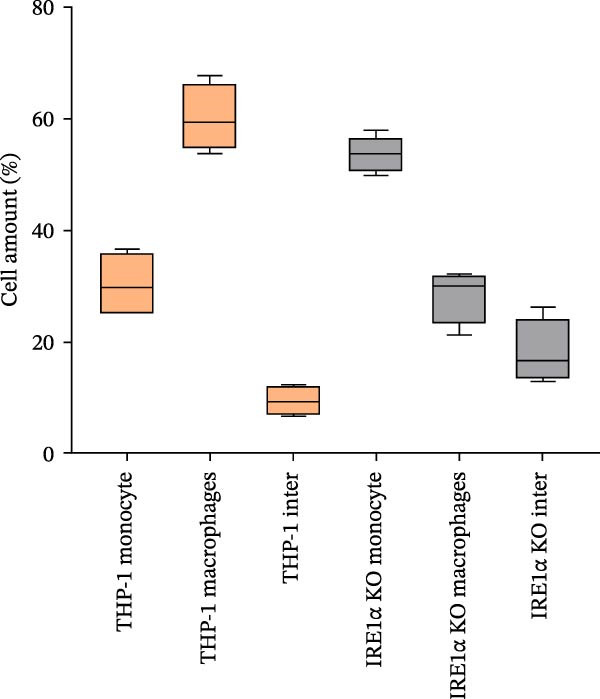
(E)

These findings demonstrate that IRE1α deficiency reduces the efficiency of monocyte‐to‐macrophage differentiation while allowing terminal differentiation to proceed in a subset of cells. In parallel, IRE1α loss is associated with stable morphological alterations present at both the monocytic and macrophage stages.

### 3.2. IRE1α Deficiency Impairs LPS‐Induced Proinflammatory Cytokine Expression

We tested a range of LPS concentrations (500, 1000, 1500, and 2000 ng/mL) to assess potential cytotoxic effects. MTT assay results demonstrated that treatment with 1000 ng/mL LPS did not affect cell viability, which remained at 100% in control cells and 99% in IRE1α KO cells (Figure S3). Based on these results, 1000 ng/mL LPS was selected for subsequent experiments. To assess the role of IRE1α in inflammatory response, we stimulated THP‐1 and THP‐1 IRE1α KO monocyte‐like cells with LPS (1000 ng/mL) for 24 h and measured proinflammatory cytokine expression.

qPCR analysis revealed that LPS treatment significantly increased gene expression of IL‐1β (4.5‐fold, *p*  < 0.0001), IL‐6 (2.6‐fold, *p*  < 0.0001), and TNF (1.65‐fold, *p*  < 0.0001) compared to unstimulated controls. In contrast, IRE1α KO monocytes exhibited severely impaired cytokine responses. IL‐1β and IL‐6 expression showed no significant increase following LPS treatment, while TNF expression was significantly reduced compared to unstimulated IRE1α KO cells (*p*  < 0.05). Importantly, direct comparison between LPS‐stimulated control group and LPS‐stimulated IRE1α KO group revealed a significant reduction in IL‐1β, IL‐6, and TNF expression in the absence of IRE1α (*p*  < 0.001 for IL‐1β, IL‐6, and TNF), demonstrating that IRE1α is required for full transcriptional activation of proinflammatory cytokines in response to LPS (Figure 3A).

Figure 3(A) Expression of IL‐1β, IL‐6, and TNF mRNA in LPS‐treated THP‐1 and THP‐1 IRE1α KO cells. THP‐1—control; THP‐1 + LPS—THP‐1 incubated with 1000 ng/mL LPS; IRE1α KO—THP‐1 with IRE1α KO; IRE1α KO + LPS—THP‐1 with IRE1α KO incubated with 1000 ng/mL LPS. (B) expression of cytokine levels IL‐1β, IL‐6, and TNF in LPS‐treated THP‐1 and THP‐1 IRE1α KO cells. Data are presented as mean ± SD. Statistical significance: ns, not significant;  ^∗^ *p*  < 0.05;  ^∗∗^ *p*  < 0.01;  ^∗∗∗^ *p*  < 0.001;  ^∗∗∗∗^ *p*  < 0.0001. One‐way ANOVA with Bonferroni correction.

**Figure:**
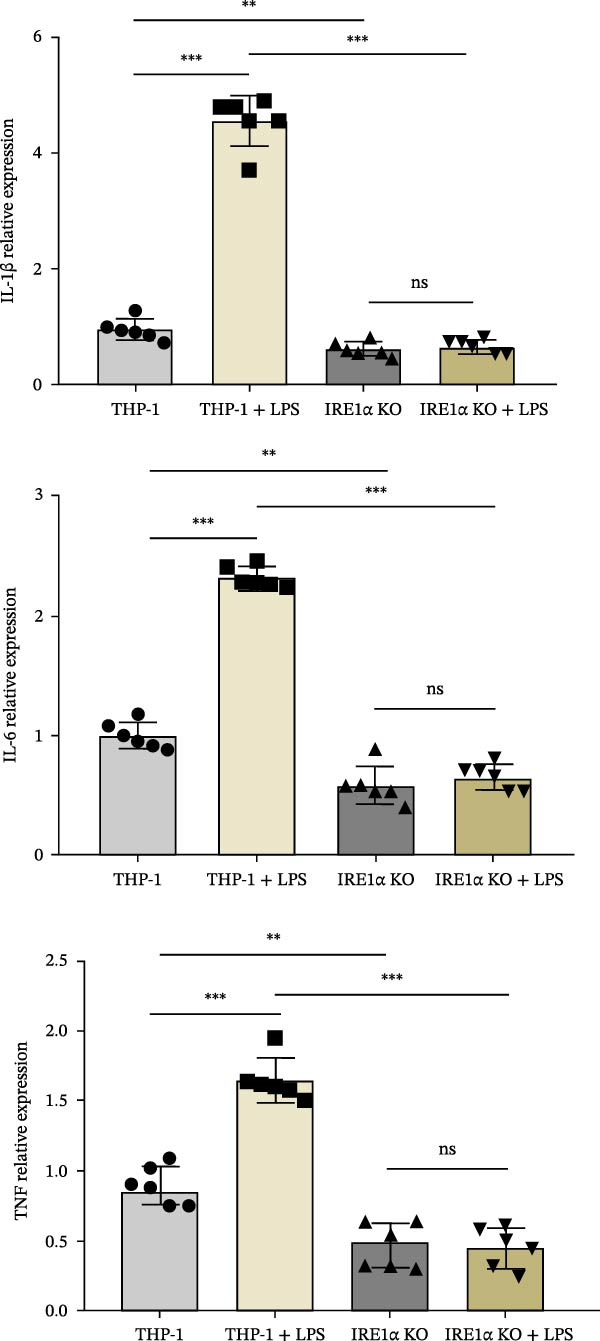
(A)

**Figure:**
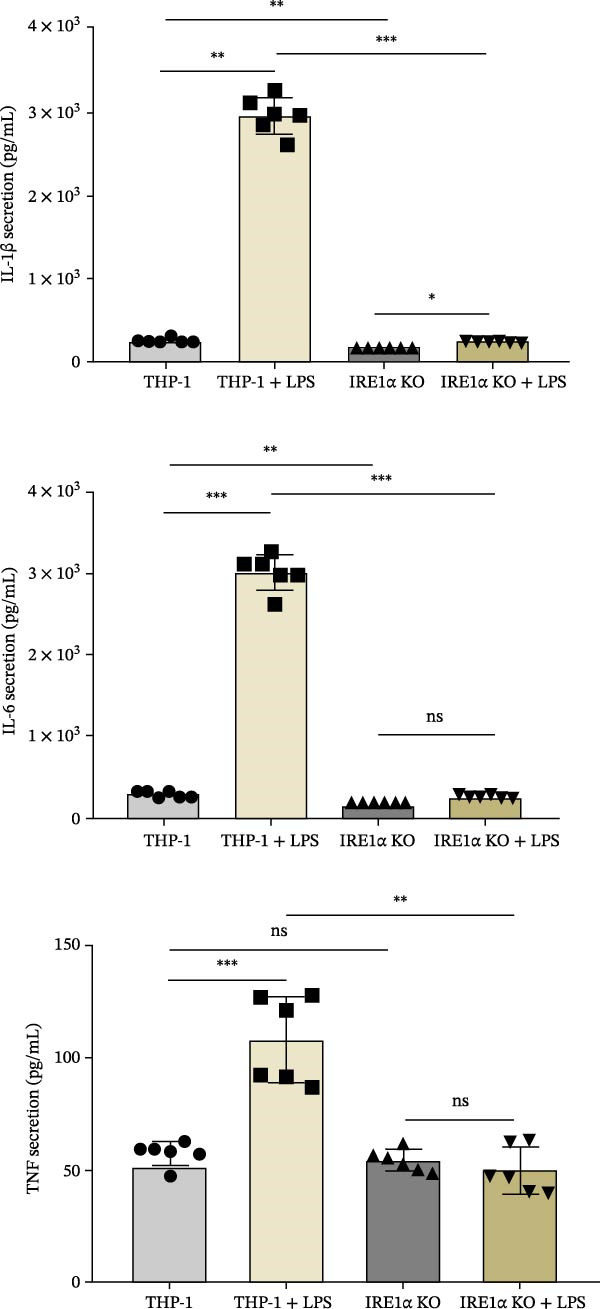
(B)

To validate these transcriptional findings at the protein level, we measured cytokine concentrations in culture supernatants using ELISA. IRE1α KO cells demonstrated significantly reduced secretion of all measured cytokines following LPS stimulation compared to control cells (Figure 3B). Specifically, TNF secretion was reduced by ~70% (*p*  < 0.001), IL‐1β by 80% (*p*  < 0.001), and IL‐6 by 75% (*p*  < 0.001) in IRE1α KO cells compared to LPS‐stimulated controls. Also, direct comparison between LPS‐stimulated control cells and LPS‐stimulated IRE1α KO cells revealed a pronounced reduction in cytokine secretion in the absence of IRE1α (Figure 3B).

Our results demonstrate that IRE1α plays an important role in mediating the proinflammatory response of monocytes. The significant upregulation of inflammatory cytokines IL‐1β, IL‐6, and TNF in THP‐1 cells following LPS stimulation underscores the importance of IRE1α in promoting these inflammatory pathways. In contrast, THP‐1 IRE1α KO cells exhibited markedly reduced expression and secretion of these cytokines upon LPS treatment, indicating that the absence of IRE1α impairs the monocyte inflammatory response.

### 3.3. The IRE1α Regulates the Intracellular Lipid Accumulation in Macrophages

To investigate the role of IRE1α in lipid‐induced inflammatory responses relevant to atherosclerosis, we examined the role of IRE1α in responses to atherogenic LDL isolated from patients with atherosclerosis. Treatment of control THP‑1 macrophage‐like cells with atherogenic LDL (100 μg/mL) for 24 h resulted in a 1.5‐fold increase in intracellular cholesterol content (*p*  < 0.001), indicating foam cell formation. In contrast, THP‐1 IRE1α KO macrophage‐like cells showed no significant cholesterol accumulation following LDL treatment (Figure 4A). Importantly, exposure to atherogenic LDL did not affect cell viability in either control or THP‐1 IRE1α KO macrophage‐like cells, as assessed by the MTT assay (Figure S1).

Figure 4(A) Comparison of total LDL cholesterol accumulation in the THP‐1 and THP‐1 IRE1α KO cell lines. THP‐1—control; THP‐1 + LDL—THP‐1 incubated with 100 μg/mL LDL; IRE1α KO—THP‐1 with IRE1α knockout; IRE1α KO + LDL—THP‐1with IRE1α knockout incubated with 100 μg/mL LDL. (B) Expression of CD36 and ABCA1 mRNA in LDL‐treated THP‐1 and THP‐1 IRE1α KO cells. Data are presented as mean ± SD. Statistical significance: ns, not significant;  ^∗^ *p*  < 0.05;  ^∗∗^ *p*  < 0.01;  ^∗∗∗^ *p*  < 0.001;  ^∗∗∗∗^ *p*  < 0.0001. One‐way ANOVA with Bonferroni correction.

**Figure:**
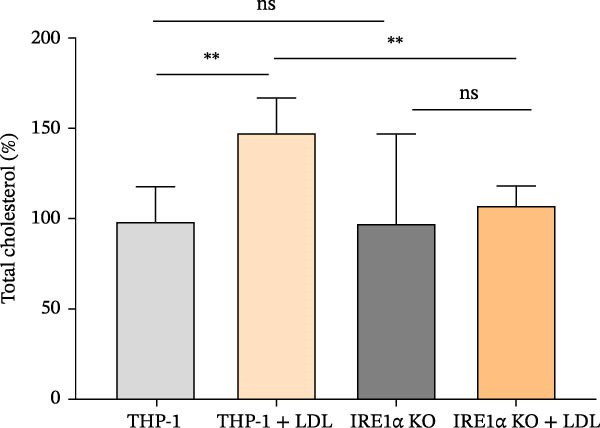
(A)

**Figure:**
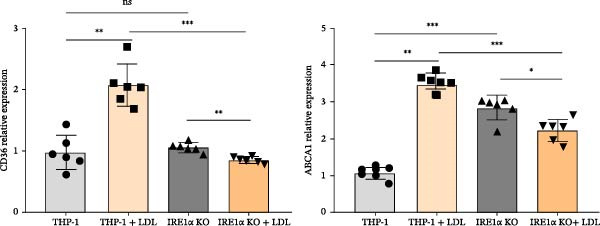
(B)

To elucidate the molecular mechanisms underlying this differential response, we performed qPCR analysis to assess expression of key genes involved in lipid metabolism: CD36, a scavenger receptor that mediates selective cholesteryl ester uptake, and ABCA1, which acts as a cholesterol efflux pump in cellular lipid removal. Analysis revealed that LDL treatment significantly upregulated CD36 (2.0‐fold, *p*  < 0.001) and ABCA1 (4.5‐fold, *p*  < 0.001) expression in control macrophage‐like cells (Figure 4B). Direct comparison between LDL‐treated control macrophage‐like cells and LDL‐treated IRE1α KO macrophage‐like cells demonstrated a significant reduction in both CD36 (*p*  < 0.001) and ABCA1 (*p*  < 0.001) expression in IRE1α KO cells (Figure 4B). However, IRE1α KO macrophage‐like cells showed reduced expression of both CD36 (1.2‐fold decrease, *p*  < 0.001) and ABCA1 (1.6‐fold decrease, *p*  < 0.001) following LDL treatment, indicating impaired lipid sensing and metabolic responses (Figure 4B). Although both CD36 and ABCA1 expression were reduced in IRE1α KO macrophage‐like cells following atherogenic LDL treatment, the net outcome was a lack of intracellular cholesterol accumulation.

Taken together, the data on the expression of the key genes involved in lipid uptake and the content of cholesterol demonstrate the role of the IRE1α gene in mediating intracellular cholesterol accumulation in response to atherogenic LDL. THP‐1 IRE1α KO cells demonstrated an inability to accumulate cholesterol upon LDL exposure, suggesting the involvement of the IRE1α gene in lipid uptake processes.

## 4. Discussion

This study demonstrates that IRE1α acts as a critical regulator of proinflammatory cytokine production in human monocytes, linking ER stress sensing to classical inflammatory pathways. Our findings provide several important insights into the molecular mechanisms underlying monocyte activation and have significant implications for understanding atherosclerotic inflammation.

We observed significant phenotypic differences in IRE1α KO cells compared to control cells. Specifically, IRE1α KO monocytes demonstrated a significant 1.7‐fold decrease in cell area compared to control cells. Similarly, IRE1α KO macrophages exhibited a 1.5‐fold decrease in median cell area relative to controls. These findings suggest a potential link between IRE1α function and cytoskeletal dynamics and correlate with the study by Urra et al. [27], wherein it was demonstrated that IRE1α regulates actin cytoskeleton dynamics and affects cell migration through a direct interaction with filamin A, an actin crosslinking factor involved in cytoskeleton remodeling. This regulation of cytoskeleton dynamics by IRE1α operates independently of its canonical role as a UPR mediator, instead functioning as a scaffold that recruits and regulates filamin A. Disruption of IRE1α‐filamin A interaction would compromise actin cytoskeleton organization, leading to the altered cell morphology and reduced cell size, as observed in our study. Furthermore, the differentiation efficiency of IRE1α KO cells into macrophages was notably lower, which is supported by UPR involvement in myeloid differentiation. A study by Tanimura et al. [28] demonstrated that UPR activity changes during neutrophil and macrophage differentiation, with IRE1α activity increasing in a stage‐specific manner throughout neutrophil differentiation. Inhibition of UPR signaling has been shown to suppress the expression of CD11b, a marker of myeloid differentiation, as well as impair morphological changes associated with differentiation, while concurrently inhibiting XBP‐1 activation [28]. Importantly, reduced differentiation efficiency does not fully account for the functional phenotype observed in IRE1α KO cells. Cells that successfully differentiated into macrophages retained canonical macrophage features, including CD68 expression and characteristic flattened morphology with vesicular cytoplasm, yet still exhibited impaired lipid uptake. This suggests that loss of IRE1α affects not only the efficiency of macrophage differentiation but also the functional competence of differentiated macrophages.

This interpretation is consistent with previous studies demonstrating that conditional deletion of IRE1α in myeloid cells does not prevent acquisition of macrophage identity markers and does not affect cell viability [29]. In addition, IRE1α has been shown to directly regulate polarization of differentiated macrophages in diverse pathological contexts, including infection and the tumor microenvironment [30].

Taken together, these findings support a model in which IRE1α contributes to regulation of cellular morphology, influences the efficiency of monocyte‐to‐macrophage differentiation, and is required for optimal functional responsiveness of mature macrophages. Given the central role of macrophages in inflammatory responses and lipid handling during atherosclerosis [31, 32], disruption of IRE1α signaling may represent a mechanistic link between ER stress, altered macrophage function, and foam cell formation in disease progression.

In response to LPS stimulation, the expression and secretion of proinflammatory cytokines TNF, IL‐1β, and IL‐6 were significantly reduced in IRE1α KO monocytes. This finding aligns with current understanding of the relationship between IRE1α and classical inflammatory pathways. IRE1α has been extensively linked to the activation of the NF‐κB transcription factor: IRE1α recruits the TRAF2 adapter, which subsequently activates the IκB kinase (IKK) complex and JNK, leading to NF‐κB activation and AP‐1 formation [33, 34]. This cascade directly regulates the transcription of proinflammatory cytokine genes, explaining the profound cytokine deficiency observed in IRE1α‐deficient monocytes. In the THP‐1 cell line, inhibition or siRNA‐mediated silencing of IRE1α significantly reduced the release of IL‐1β, IL‐6, and TNF following stimulation with LPS or LPS combined with nigericin [35]. Corresponding results were observed in animal models, where IRE1α‐deficient mice exhibited decreased levels of IL‐1β, IL‐6, and TNF in neutrophils and monocytes, along with reduced tissue concentrations of these cytokines [36]. These findings indicate that the IRE1α/XBP1‐dependent pathway plays a critical role in enhancing the production of key inflammatory cytokines. Also, we should note the remarkable effect on IL‐1β production in IRE1α KO cells. Previous studies have demonstrated that IRE1α links with NLRP3 inflammasome activation [37]. This mechanism may involve IRE1α‐dependent expression of TXNIP, a protein that facilitates inflammasome assembly. Our findings suggest that IRE1α deficiency disrupts optimal pro‐IL‐1β expression and/or impairs inflammasome activation, resulting in decreased IL‐1β secretion. The impaired cytokine responses in IRE1α KO cells have important implications for atherosclerosis, where these specific cytokines play well‐established pathogenic roles. TNF promotes endothelial activation and expression of adhesion molecules, facilitating monocyte recruitment [38]. IL‐6 drives acute‐phase responses and contributes to systemic inflammation associated with cardiovascular risk [39]. IL‐1β is particularly relevant to atherosclerosis, as demonstrated by the success of IL‐1β inhibition (canakinumab) in reducing cardiovascular events in clinical trials [40].

Our observation that IRE1α deficiency prevents foam cell formation and lipid accumulation provides additional evidence for its role in atherosclerotic processes. The inability of IRE1α KO macrophage‐like cells to upregulate CD36 in response to atherogenic LDL suggests that IRE1α is essential for sensing lipid overload and initiating appropriate transcriptional responses. This finding is particularly relevant given that CD36 is a key receptor for oxidized LDL uptake and foam cell formation [41]. In this study, we used patient‐derived atherogenic LDL rather than artificially oxidized LDL to better reflect physiologically relevant lipoprotein–macrophage interactions. LDL isolated from patients with atherosclerosis carries a heterogeneous spectrum of in vivo modifications, including oxidative and nonoxidative alterations, which collectively determine macrophage uptake and foam cell formation more accurately than in vitro‐generated oxLDL [42, 43]. The cholesterol accumulation observed in control macrophages supports that this LDL preparation is functionally atherogenic in vitro and supports the relevance of our experimental model. This functional definition of atherogenic LDL is consistent with previous studies demonstrating that macrophage cholesterol accumulation and foam cell formation represent a more physiologically relevant measure of LDL atherogenicity than isolated biochemical indices of oxidation. LDL‐induced foam cell formation is a central event in atherogenesis and has been widely used as a functional readout of LDL atherogenic potential in cellular systems [44]. Moreover, patient‐derived atherogenic LDL species that stimulate unregulated uptake and intracellular lipid accumulation by macrophages have been shown to drive foam cell formation independently of simple oxidation status, indicating that multiple in vivo modification pathways contribute to LDL atherogenicity beyond oxidation alone [45]. Notably, IRE1α deficiency resulted in a coordinated downregulation of ABCA1 expression, highlighting a complex deregulation of cholesterol efflux mechanisms in addition to impaired lipid uptake. Although ABCA1 downregulation is typically considered proatherogenic due to its essential role in reverse cholesterol transport and HDL biogenesis [46, 47]. However, its functional impact in our experimental context appears limited. In control THP‐1 macrophage‐like cells, exposure to patient‐derived atherogenic LDL for 24 h increased intracellular cholesterol and induced the expression of both CD36 and ABCA1 (Figure 4A, B), consistent with parallel induction of uptake‐associated and compensatory efflux‐associated transcriptional programs during early lipid loading. In contrast, IRE1α KO macrophage‐like cells failed to upregulate CD36 and ABCA1 in response to modified LDL and did not accumulate cholesterol over the same time window (Figure 4A, B). CD36 is a principal scavenger receptor mediating macrophage uptake of modified LDL and contributing to lipid loading and foam cell formation.

At the same time, ABCA1 is generally considered atheroprotective because it mediates cholesterol and phospholipid efflux to lipid‐poor apoA‐I, initiating HDL biogenesis and supporting macrophage cholesterol efflux/reverse cholesterol transport; accordingly, sustained ABCA1 suppression would be expected to impair cholesterol removal and could be detrimental in a chronic plaque setting [48–50]. Mechanistically, ABCA1 is a canonical transcriptional target of the oxysterol‐activated LXR/RXR pathway and is typically induced upon cholesterol loading as a compensatory response to reduce intracellular cholesterol burden [51, 52]. In addition, modified lipid ligands can activate PPARγ, which promotes scavenger receptor expression (including CD36) and can also drive a PPARγ→LXRα→ABCA1 transcriptional cascade that couples lipid uptake to cholesterol efflux programs [53, 54]. Therefore, we interpret the concomitant reduction of CD36 and ABCA1 in IRE1α KO macrophage‐like cells as an uptake‐limited phenotype during the acute (24 h) LDL‐loading phase: impaired CD36 induction likely restricts lipid influx and downstream intracellular cholesterol/oxysterol signals that normally activate compensatory LXR‐dependent efflux pathways (including ABCA1). This framework reconciles the reduced net cellular cholesterol content at 24 h with the established protective role of ABCA1, and it highlights that the biological consequences of ABCA1 downregulation are expected to be strongly time‐ and context‐dependent (acute loading versus chronic lipid handling).

Because our study assessed mRNA expression and total cellular cholesterol at a single time point, future work will be needed to quantitatively disentangle uptake versus efflux contributions, including direct functional assays of LDL uptake and apoA‐I/HDL‐dependent cholesterol efflux across a time course, as well as confirmation at the protein level for CD36 and ABCA1.

Our findings identify IRE1α as a potential therapeutic target for modulating macrophage‐driven inflammation. Several small‐molecule IRE1α inhibitors have been developed, including compounds targeting its kinase activity (KIRA6) or RNase activity (4μ8C, STF‐083010) [55, 56]. Given our results, these inhibitors might reduce proinflammatory cytokine production in atherosclerotic plaques while simultaneously preventing foam cell formation. However, therapeutic targeting of IRE1α requires careful consideration of its physiological roles. Complete IRE1α inhibition might compromise adaptive UPR functions essential for cellular survival under stress conditions. Future studies should investigate whether partial IRE1α modulation can achieve anti‐inflammatory benefits while preserving protective UPR functions.

## 5. Conclusion

The results presented in this study demonstrate a pivotal role of IRE1α in regulating proinflammatory cytokine production in human monocytes, linking ER stress sensing to classical inflammatory pathways including NF‐κB and inflammasome activation. IRE1α KO attenuates LPS‐induced TNF, IL‐1β, and IL‐6 expression and secretion, while simultaneously preventing lipid‐induced foam cell formation. These findings identify IRE1α as an attractive therapeutic target for modulating macrophage‐driven inflammation in atherosclerosis and potentially other chronic inflammatory diseases. The dual function of IRE1α in both inflammatory cytokine regulation and lipid metabolism positions it as a central regulatory hub in atherosclerotic pathogenesis, where metabolic stress and inflammation are mechanistically interconnected. Our results demonstrate that IRE1α coordinates both the uptake of modified lipoproteins and the subsequent inflammatory response, making it an ideal target for therapeutic intervention. Future therapeutic strategies targeting IRE1α should aim to achieve optimal anti‐inflammatory effects while preserving essential adaptive UPR functions, potentially through selective inhibition approaches or temporal modulation strategies.

## Author Contributions

Mariam Bagheri Ekta, Vasily Sukhorukov, and Stanislav Antonov contributed to the conceptualization and study design. Mariam Bagheri Ekta wrote the main manuscript text. Vasily Sukhorukov and Stanislav Antonov reviewed and edited the manuscript text. Mariam Bagheri Ekta and Natalia Elizova performed the experiments. Mariam Bagheri Ekta, Vasily Sukhorukov, and Natalia Elizova carried out data curation. Stanislav Antonov prepared Figure 2. Mariam Bagheri Ekta prepared Figures 1, 3, and 4. Alexander Orekhov provided funding. Vasily Sukhorukov supervised the study.

## Funding

This work was supported by the Russian Science Foundation (Grant # 25‐15‐00483).

## Disclosure

All authors have read and agreed to the published version of the manuscript.

## Conflicts of Interest

The authors declare no conflicts of interest.

## Supporting Information

Additional supporting information can be found online in the Supporting Information section.

## Supporting information


**Supporting Information** Figure 1. Effect of patient‐derived atherogenic LDL on THP‐1 and THP‐1 IRE1α KO macrophage‐like cells viability. Cell viability of control THP‐1 macrophage‐like cells (A) and THP‐1 IRE1α KO macrophage‐like cells (B) following 24 h treatment with atherogenic LDL was assessed using the MTT assay. No significant differences were observed between groups. Supporting figure 2. Differentiation of monocytes into macrophages in THP‐1 and IRE1α KO cells. The figure depicts three distinct cell types: 1—monocyte; 2—macrophage; 3—intermediate form between monocytes and macrophages. Each cell type is labeled with corresponding numbers for clarity. The morphological features of these cells were analyzed to assess the differentiation process. Supporting Figure 3. Effect of increasing LPS concentrations on the viability of THP‐1 and THP‐1 IRE1α knockout monocytes. Cell viability of control THP‐1 cells (A) and THP‐1 IRE1α KO cells (B) following 24 h stimulation with increasing concentrations of LPS (500–2000 ng/mL) was assessed using the MTT assay. No significant reduction in viability was observed at 500–1000 ng/mL, whereas higher concentrations induced cytotoxicity.

## Data Availability

The data that support the findings of this study are available from the corresponding author upon reasonable request.
