# Supplementary material for: The IRE1α Pathway Links Endoplasmic Reticulum Stress to Atherosclerosis‐Related Inflammation and Lipid Accumulation
Source: Mediators Inflamm. 2026 Apr 8;2026:4439938. doi: 10.1155/mi/4439938 (PMC13058818; doi:10.1155/mi/4439938)
Supplement: Supplementary file 1 — Supporting Information Figure 1. Effect of patient‐derived atherogenic LDL on THP‐1 and THP‐1 IRE1α KO macrophage‐like cells viability. Cell viability of control THP‐1 macrophage‐like cells (A) and THP‐1 IRE1α KO macrophage‐like cells (B) following 24 h treatment with atherogenic LDL was assessed using the MTT assay. No significant differences were observed between groups. Supporting figure 2. Differentiation of monocytes into macrophages in THP‐1 and IRE1α KO cells. The figure depicts three distinct cell types: 1—monocyte; 2—macrophage; 3—intermediate form between monocytes and macrophages. Each cell type is labeled with corresponding numbers for clarity. The morphological features of these cells were analyzed to assess the differentiation process. Supporting Figure 3. Effect of increasing LPS concentrations on the viability of THP‐1 and THP‐1 IRE1α knockout monocytes. Cell viability of control THP‐1 cells (A) and THP‐1 IRE1α KO cells (B) following 24 h stimulation with increasing concentrations of LPS (500–2000 ng/mL) was assessed using the MTT assay. No significant reduction in viability was observed at 500–1000 ng/mL, whereas higher concentrations induced cytotoxicity. [file MI-2026-4439938-s001.docx]

**Supplementary Figure 1.** Effect of patient-derived atherogenic LDL on THP-1 and THP-1 IRE1α KO macrophage-like cells viability. Cell viability of control THP-1 macrophage-like cells (A) and THP-1 IRE1α KO macrophage-like cells (B) following 24 h treatment with atherogenic LDL was assessed using the MTT assay. No significant differences were observed between groups.


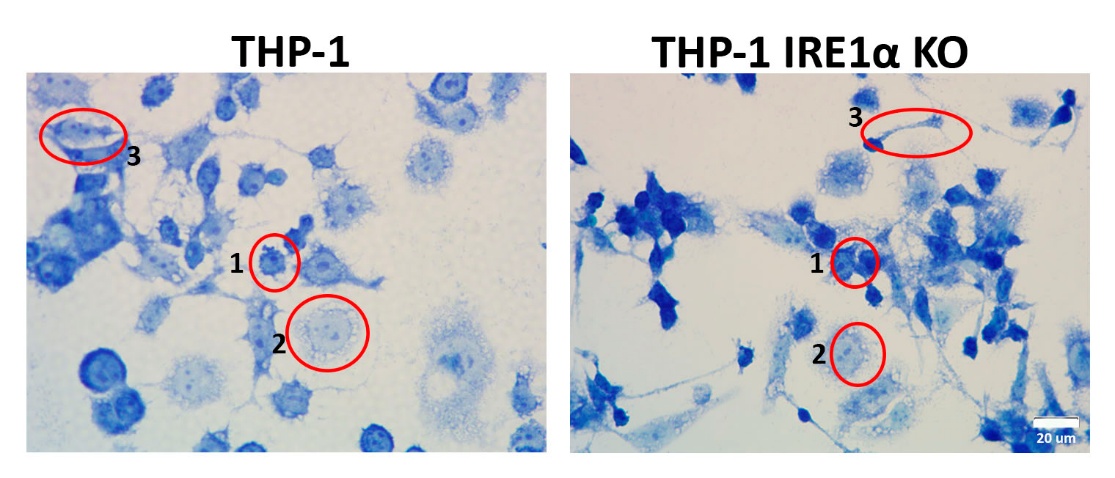


**Supplementary figure 2.** Differentiation of monocyte into macrophages in THP-1 and THP-1 IRE1α knockout cells. The figure depicts three distinct cell types: 1 – monocyte; 2 – macrophage; 3 – intermediate form between monocytes and macrophages. Each cell type is labeled with corresponding numbers for clarity. The morphological features of these cells were analyzed to assess the differentiation process.

**Supplementary Figure 3.** Effect of increasing LPS concentrations on the viability of THP-1 and THP-1 IRE1α knockout monocytes. Cell viability of control THP-1 cells (A) and THP-1 IRE1α knockout cells (B) following 24 h stimulation with increasing concentrations of LPS (500–2000 ng/mL) was assessed using the MTT assay. No significant reduction in viability was observed at 500–1000 ng/mL, whereas higher concentrations induced cytotoxicity.
